# Supplementary material for: Reconstructing phase-resolved hysteresis loops from first-order reversal curves
Source: Sci Rep. 2021 Feb 17;11:4018. doi: 10.1038/s41598-021-83349-z (PMC7889904; doi:10.1038/s41598-021-83349-z)
Supplement: Supplementary file 1 — Supplementary information. [file 41598_2021_83349_MOESM1_ESM.docx]

**Supplemental Material for:**

**Reconstructing phase-resolved hysteresis loops from first-order reversal curves**

Dustin A. Gilbert,^1, 2 *^ Peyton D. Murray,^3^ Julius De Rojas,^3^ Randy K. Dumas,^4^ Joseph E. Davies,^5^ Kai Liu^3, 6^

*^1^ Department of Materials Science and Engineering, University of Tennessee, Knoxville, Tennessee 37919*

*^2^ Department of Physics and Astronomy, University of Tennessee, Knoxville, Tennessee 37919*

*^3^ Physics Dept., University of California, Davis, CA 95616*

*4 Quantum Design, Inc., San Diego, California 92121*

*^5^ Advanced Technology Group, NVE Corp., Eden Prairie, MN, 55344*

*^6^ Department of Physics, Georgetown University, Washington, D.C. 20057*

FORC measurements were performed on a commercially available 2 mm diameter Y_3_Fe_2_(FeO_4_)_3_ (YIG) calibration standard purchased from the National Institute of Standards and Technology. This sample has a saturation moment, *M_S_*, of 75.955 memu (1 memu = 1 μA·m^2^) and a saturation field of 65 mT. The major loop and family of FORCs are shown in Fig. S1, with the FORC distribution (b) clipped and treated with (c) constant and (d) linear extensions.

The clipped data, Fig. S1(b), shows no features, consistent with complete suppression of the reversible behavior. By comparison, the constant extended data, Fig. 2(c), shows a single positive feature at H_C_=0, oriented parallel to the H_B_ axis, and centered at H_B_=0, with a spread in H_B_ of ±62.5 mT, in agreement with the saturation field from the major loop. The linear extension, Fig. 2(d), shows a positive negative feature pair occurring at the endpoints of the vertical feature seen in Fig. 2(b). Specifically, at H_C_=0, for dρ/dH_B_ < 0 in the constant extension, panel (c), a positive feature manifests, and for dρ/dH_B_ < 0 a negative feature manifests. Thus we observe, even for this simple case, that the different approaches to extending the dataset cause significant qualitative changes to the FORC distribution.

Integrating the FORC distribution give a quantitative insight to these changes. The integral of the FORC distribution from the clipped data gives a $M_{S}^{Clip.}$ of 22.1 μemu (0.005 M_S_), while the integral of the constant extended data gives $M_{S}^{Const.}$ of 76.5 memu (1.007 M_S_), and the linear extended data $M_{S}^{Lin.}$ of 26.5 μemu (0.006 M_S_). Thus clipping the data or using a linear extension is shown to quantitatively suppress the reversible contribution. For the case of clipping the reversible feature is rudimentarily removed from the FORC distribution, while the linear extension realizes a positive negative feature pair which are of equal and opposite weight. By comparison, the constant extension accurately recovers the major loop *M_S_* and saturation field. This simple case suggests that clipping the data or applying a linear extension can quantitatively reject the reversible component, while a constant extension accurately manifests a feature representative of the reversible component.


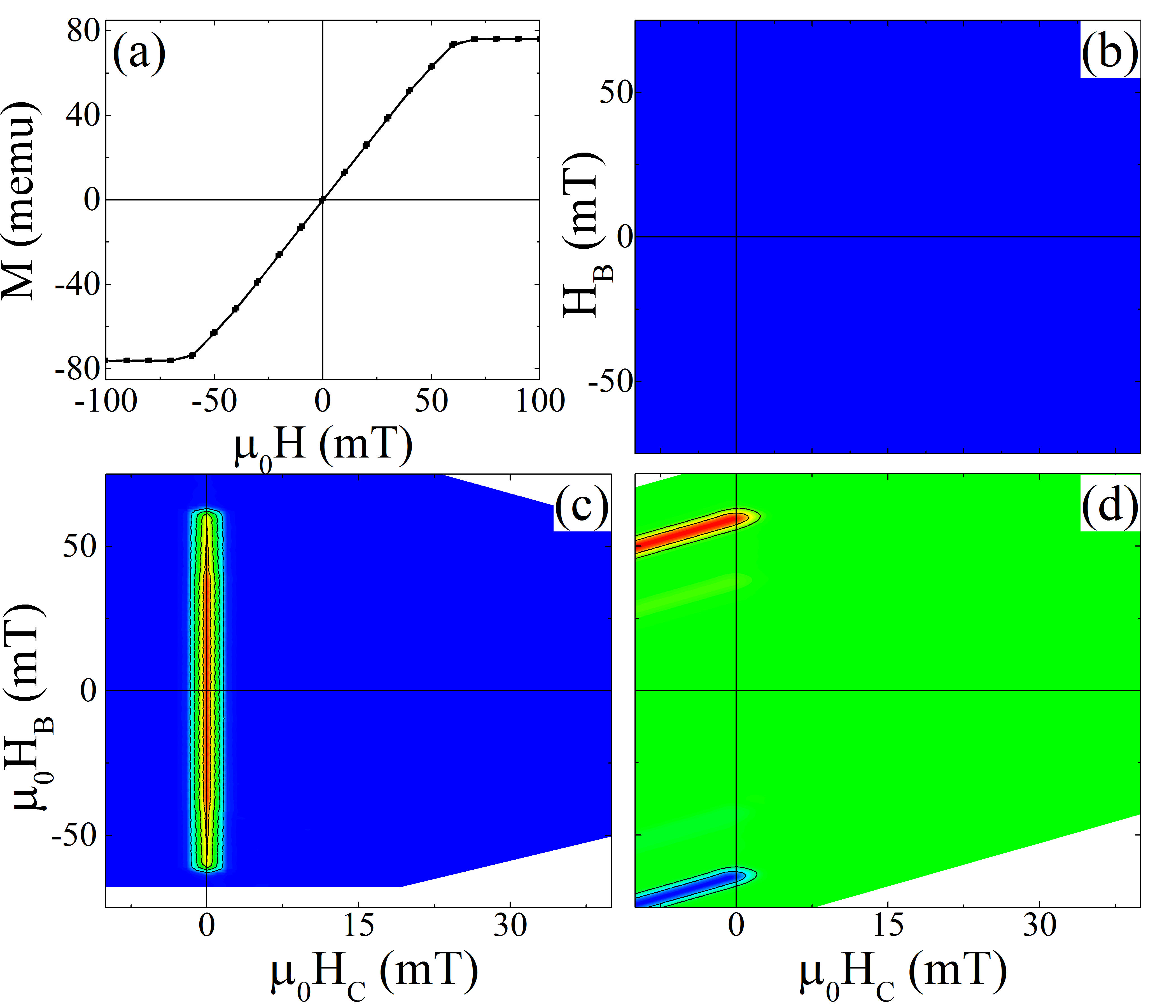


**Figure S1** (a) Major hysteresis loop and (b-d) FORC diagrams for a fully-reversible YIG sphere. The FORC distributions demonstrate (b) clipping, (c) a constant extension and (d) a constant slope extension.
